# The Impact of COVID-19 in Brazil Through an Educational Neuroscience Lens: A Preliminary Study

**DOI:** 10.3390/brainsci15060548

**Published:** 2025-05-23

**Authors:** Camila G. Fonseca, Camila L. L. Dias, Marcus L. L. Barbosa, Maria Julia Hermida, Luiz Renato R. Carreiro, Alessandra G. Seabra

**Affiliations:** 1 Post-Graduation Program in Human Development Sciences, CCBS—Mackenzie Presbyterian University, Rua da Consolação, nº 930, São Paulo 01302-907, SP, Brazil; camilafonsecapsi@gmail.com (C.G.F.); camila.cldias@gmail.com (C.L.L.D.); luizrenato.carreiro@mackenzie.br (L.R.R.C.); 2 Post-Graduation Program in Cultural Diversity and Social Inclusion, Feevale University, ERS-239, 2755, Novo Hamburgo 93525-075, RS, Brazil; marcusl@feevale.br; 3 Instituto de Educación, Universidad Nacional de Hurlingham, Vergara 2222, Villa Tesei B1688, Provincia de Buenos Aires, Argentina; julia.hermida@gmail.com

**Keywords:** executive functioning, academic performance, reading, writing, COVID-19 pandemic

## Abstract

**Background:** Educational neuroscience has made important contributions to show how the COVID-19 pandemic impacted schooling. In countries like Brazil, with significant educational inequality, the suspension of in-person classes worsened these disparities, as low-income families faced difficulties accessing remote learning. **Methods:** This study evaluated executive functions (EF) and academic skills in reading, writing, and maths for 178 public school students from the first to ninth grades in São Paulo, Brazil, comparing them with pre-pandemic norms to assess possible differences. EF were assessed using the Hayling Test, Digit Span Task, and Verbal Fluency, while academic skills were measured by the School Performance Test II. To analyse differences between the sample of this study and the pre-pandemic normative samples, one-sample t-tests were performed. Due to the small sample size, segmented by school grade and age, the bootstrapping resampling method was used, and the effect size was measured with Cohen’s d. **Results:** A one-sample *t*-test showed significant differences between times, with lower post-pandemic performance in verbal fluency (9–14 years old), working memory (10–14 years old), and inhibitory control across all age groups. Writing skills were lower from the fifth to eighth grades and reading from the fourth to eight grades. Maths skills were lower in the fourth, eighth, and ninth grades. Better post-pandemic performance was seen in working memory (6 and 7 years old). **Conclusions:** Students in the upper grades of elementary school during the pandemic were most impacted by the suspension of in-person teaching, highlighting the importance of schooling and the need for recovery efforts at these levels.

## 1. Introduction

The COVID-19 pandemic and the consequent disruption of in-person education were among the major recent events with a strong impact on schooling [1]. The impact was not only due to lack of schooling but also because in the cases in which schooling continued in a virtual form, the quality of education was not guaranteed. For example, Engzell et al. [2] found that students made little or no progress in learning during the closure. Specifically, Engzell et al. [2] found that in only eight weeks of school closure, Dutch primary students lost the equivalent of one-fifth of a school year, with disadvantaged households suffering disproportionately—suggesting cumulative deficits in knowledge acquisition tied to reduced in-person learning. These findings are in line with global trends reported in the literature. The meta-analysis conducted by König and Frey [3] highlighted robust negative effects, particularly among younger students, indicating heightened vulnerability during critical developmental windows. Additionally, a systematic review and meta-analysis conducted in 2022 [4], which assessed the scientific production of 15 countries, revealed that students, during the pandemic, experienced an average loss of 35% of the content corresponding to a school year. Broader evidence from PISA data [5] quantified mathematics losses at 14% of a standard deviation (approximately 7 months), disproportionately affecting marginalised groups and correlating with closure duration. Logically, children without access to the internet and a large-screen device, with poorly educated parents, and without a quiet space to study were more affected [6]. In consequence, school closures in all countries are likely to have sharpened existing disparities as socioeconomic status is positively associated with children’s ability to adjust to remote learning [7].

Notably, Lynch et al. [8] observed mixed outcomes in Head Start preschoolers, with modest executive function (EF) gains (0.05 SD) but stronger progress in numeracy (0.45–0.71 SD), suggesting domain-specific resilience for neurocognitive skill development. Framed within educational neuroscience, these results highlight the pandemic’s uneven impact on neurocognitive systems: while executive function (linked to prefrontal maturation) showed limited growth in the absence of in-person scaffolding [8], academic skills such as numeracy may be more responsive to targeted interventions.

The collective evidence underscores the need for studies addressing the impact of the pandemic on EF as well as academic outcomes. For that objective, educational neuroscience has proven crucial in identifying the neurocognitive effects of schooling disruptions and in defining potential targets for intervention. In fact, during remote teaching, educational neuroscience contributed to the exploration of educational practices [9], the improvement of educational practices [10], teacher training [11], and the measurement of the impact of educational disruption on neurodevelopment [12].

Specifically in Brazil, an Emergency Remote Learning model was implemented, and about 5.5 million Brazilian children had limited or no access to education during this period [13]. Brazil was one of the countries where schools remained closed the longest, with an average of 279 days closed in 2020 and an additional 103 days in 2021 [14]. Furthermore, 38% of students (OECD average: 34%) had difficulty understanding school assignments at least once a week, and about 30% (OECD average: 24%) struggled to find someone to help them with schoolwork [15].

In Brazil, three types of approaches were implemented to evaluate the impact of the pandemic. First, an analysis of national standardised academic achievement test scores obtained after the closure ended indicated that the greatest drop occurred in written language in the second year of elementary school, where the average score fell from 750 points in 2019 to 725.9 points in 2021, about half a standard deviation. In mathematics, there was also a decline, though less significant, from 750 to 741.6 points. In later school years, the biggest declines occurred in mathematics, with fifth- and ninth-grade students showing score drops of 11 and 7 points, respectively, compared to 2019. In language, there was a drop of 6.6 points for fifth graders and 2.2 points for ninth graders [14,16].

A second group of correlational studies analysed which ages were most impacted. For example, Alves et al. carried out a longitudinal study with students from the second to the fifth grade of elementary school in Minas Gerais [17], aiming to assess reading fluency during the remote learning period. The findings indicated that second-grade students suffered the greatest negative impact due to school closures and achieved lower results in March 2022 compared to the same period in the previous year (the beginning of the COVID-19 pandemic). This result suggests that the younger the children the most impacted their academic achievement. This is possibly because older students, in the final years of elementary school or in high school, seem to be more familiar with technology and, therefore, are more autonomous [18,19,20].

A third type of study addressed the impact of the pandemic, comparing the pre-pandemic norms with the post-pandemic scores obtained by high-socioeconomic-status children. For example, in a study conducted with children in grades 1 to 5 of a private elementary school in a city of medium to high socioeconomic status in the interior of São Paulo state [21], the results showed significantly lower performance compared to the pre-pandemic normative sample. The declines were observed in arithmetic for third, fourth, and fifth grades and in writing for second, fourth, and fifth grades.

To worsen the situation, in addition to the deficits observed in academic learning, some studies have also suggested cognitive deficits, specifically in executive functions (EF), in various countries [22,23,24]. In Brazil, Campos, Seabra, and Carreiro longitudinally followed students in the early years of elementary school before and during the pandemic, at the beginning and end of 2020, and observed an increase in complaints about EF-related problems among students [25]. In another Brazilian study, after schools reopened, 117 students from the first and second grades in a public school had their neuropsychological functions assessed. The results pointed to a high prevalence of children in the literacy phase showing alert states or deficits in orientation, memory, attention, language, visuospatial skills, arithmetic skills, and verbal fluency [26]. However, there are still a few studies in Brazil aimed at describing potential EF and academic losses across different school years. This preliminary study aimed to contribute to mapping executive functioning and reading, writing, and math skills in Brazilian students from the first to ninth grades after the pandemic, comparing them with the pre-pandemic normative data of the assessment instruments used to determine possible performance discrepancies. Based on the above-mentioned studies, we expect to find a decrease in EF as well in academic achievement across all grades levels, with respect to the pre-pandemic norms.

## 2. Materials and Methods

### 2.1. Participants

Initially, 193 elementary school children from three public schools in two cities of the Greater São Paulo area participated in the study, with 82 students from the city of Santo André and 111 students from the city of Embu das Artes. Given the preliminary nature of this study, a non-probabilistic sample was chosen, selected for convenience. A post hoc power analysis was conducted to determine the statistical power of the final sample (n = 178) to detect effects. For a one-sample t-test (comparing means to a reference value) with a moderate effect size (Cohen’s d = 0.5) and α = 0.05 (two-tailed), the achieved power was > 99% (calculated using G*Power 3.1). Even for a smaller effect size (d = 0.2), the power remained high (85%), indicating robust sensitivity to detect clinically meaningful differences. All children from these schools were invited to participate; however, certain diagnoses were used as exclusion criteria based on school records, including attention deficit hyperactivity disorder, intellectual disability, autism spectrum disorder, and specific learning disorder.

Subsequently, after analysing the results, participants with an intellectual level potentially indicative of undiagnosed intellectual disability were excluded. Specifically, exclusion criteria included scores below the 5th percentile on the Raven’s Colored Progressive Matrices test, for participants aged 6 to 10 years, and an intelligence quotient below 70, according to results from the Wechsler Abbreviated Scale of Intelligence [WASI], for participants aged 11 and older. Based on this criteria, fifteen participants were excluded from the sample.

The participants’ ages ranged from 6 to 14 years (M = 10; SD = 5.65), with 55.62% female and 44.38% male. Regarding grade level, 8.44% were in the 1st grade, 10.11% in the 2nd and 3rd grades, 12.92% in the 4th grade, 14.04% in the 5th grade, 12.36% in the 6th grade, 11.80% in the 7th grade, 10.67% in the 8th grade, and 9.55% in the 9th grade.

Data collection occurred in the second semester of 2023 (from September to November) and the first semester of 2024 (from May to July). Table 1 presents the school grade of the study participants during the pandemic period (2020 and 2021) and at the time of data collection (2023 and 2024).

The city of Santo André has an average monthly salary for formal workers of approximately USD 625.92 [27]. In the 2023 Basic Education Development Index (IDEB), the city scored 6.5 out of 10 points in the early years of elementary education in public schools and 5.2 out of 10 points in the final years [14]. Embu das Artes has an average monthly salary for formal workers of approximately USD 650.03 [27]. The IDEB for the early years of elementary education in public schools is 6.2 out of 10 points in the early years and 5.0 out of 10 points in the final years [14]. Both cities have IDEB close to the average for the State of São Paulo (6.2 in the initial grades and 5.1 in the final grades).

In March 2020, in-person classes were suspended for all children. Regular school attendance was resumed in February 2022, following government guidelines.

### 2.2. Instruments

The following instruments were used to evaluate child cognitive performance:

- Digit Span Task (DST): Assesses short-term verbal memory (forward order—FO) and working memory (backward order—BO). The examinee’s task is to repeat sequences of digits in either forward or backward order. The test was administered individually, and the measures used were total correct responses (sum of both orders). This task is standardised for the Brazilian population, and there is evidence of validity for ages 4 to 10 years [28]. In this study, this instrument was used with children up to 10 years old. For this task, the minimum possible raw score is 0, and the maximum possible raw score is 16.

- Digit Span Subtest from the Wechsler Intelligence Scale for Children—WISC IV: Assesses short-term verbal memory (forward order—FO) and working memory (backward order—BO). The examinee’s task is to repeat sequences of digits in either forward or backward order. The test was administered individually, and the measures used were total correct responses (sum of both orders). This task is standardised for the Brazilian population and has demonstrated validity for individuals aged 6 years to 16 years and 11 months [29]. In this study, this instrument was used with children aged 11 and older. For this task, the minimum possible raw score is 0, and the maximum possible raw score is 16.

- Free Verbal Fluency (FVL): Assesses linguistic-mnemonic processes related to lexical search and components of executive functions, such as cognitive flexibility, inhibition, working memory, and search strategy organisation. In this individually administered task, the examinee must say as many words as possible within a given time (two and a half minutes), respecting the guidelines to keep their eyes closed and not say proper names or numbers. This task is standardised for the Brazilian population, and its validity was probed with individuals aged 6 to 12 years [30]. In this study, for participants older than the normative sample, data corresponding to age 12 were used only to determine whether, even when compared to younger children, there could be indicators of difficulty in these cognitive skills. For this instrument, the minimum possible raw score is zero, and the maximum raw score is equivalent to the number of correct answers.

- Hayling Test (HT): The HT is divided into two parts, A and B, which assess, respectively, linguistic aspects and EF (such as cognitive flexibility and inhibitory control), as well as processing speed. In this individually administered task, the participant must, in Part A, complete the sentences with words that fit appropriately in the context, as quickly as possible. In Part B, the objective is to complete the sentences with words unrelated to the context presented. There are two versions, both with evidence of validity and standardisation for the Brazilian population: Children’s Hayling Test (CHT), used with individuals aged 6 to 12 years [31], and Adult’s Hayling Test (AHT), from age 13 onwards [32]. Part A is only a basal analysis to assess EF in Part B; in this study, we used only measures related to Part B. The measures used were execution time in Part B, which is the sum of the reaction time in seconds of all items, as well as the number of errors in Part B. For CHT, the minimum possible raw score is 0, and the maximum possible raw score is 10, but for AHT, the minimum possible raw score is 0, and the maximum possible raw score is 15.

School achievement was evaluated with the following instruments:

- School Performance Test II (TDE II): The TDE II aims to assess basic skills in reading, writing, and arithmetic. There is evidence of validity for this test, which has also been standardised for the Brazilian population, with students from the first to the ninth years of elementary school [33]. Below is a brief description of each part:

- Writing Subtest: Consists of a list of 40 words that are read one by one to the students, and they must write them down. There is one version for the 1st to 4th grades and another for the 5th to 9th grades. The test was administered collectively, with time being measured, and scoring was interrupted after 10 consecutive errors or “don’t know” responses. The measures used were the score and the duration of the task. For this task, the minimum possible raw score is 0, and the maximum possible raw score is 40, in both versions.
- Reading Subtest: Composed of a list of 36 words for the 1st to 4th grades, and 33 words for the 5th to 9th grades, which the examinee must read aloud individually. The time was recorded, and scoring was interrupted after 10 consecutive errors or “don’t know” responses. The measures used were the score and the duration of the task. For students in grades 1 to 4, the minimum possible raw score is 0, and the maximum possible raw score is 36, but for students in grades 5 to 9, the minimum possible raw score is 0, and the maximum possible raw score is 33.
- Arithmetic Subtest: Consists of a list of 37 exercises for the 1st to 5th grades, and 43 for the 6th to 9th grades, which the examinee must solve. The test was administered individually for children from the 1st to 3rd grades and collectively for other participants, according to the manual’s guidelines. The time was recorded, and scoring was interrupted after 10 consecutive errors or “don’t know” responses. The measures used were the score and the duration of the task. For students in grades 1 to 5, the minimum possible raw score is 0, and the maximum possible raw score is 37, but for students in grades 6 to 9, the minimum possible raw score is 0, and the maximum possible raw score is 43.

To check inclusion criteria, we administered the following tests:

- Raven’s Coloured Progressive Matrices (CPM): The CPM assesses general intelligence through visual puzzles designed to evaluate pattern recognition. It is organised in three parts, which are arranged in increasing difficulty. There is evidence of validity, and it was standardised for the Brazilian population, specifically for individuals aged 5 to 11 years [34]. In this study, this instrument was administered to children from the 1st to 5th grades to screen for significant intellectual deficits as an exclusion criterion.

- Wechsler Abbreviated Scale of Intelligence (WASI): This is a brief intelligence assessment tool. The Vocabulary and Matrix Reasoning subtests were individually administered to estimate general cognitive functioning. There is evidence of the validity for this test, which is also standardised for the Brazilian population, with normative study covering individuals aged 6 to 89 years [35]. In this study, it was used for children from the 6th grade onwards to screen for significant intellectual deficits as exclusion criteria.

### 2.3. Data Collection Procedures

The project was approved by the Research Ethics Committee of the proposing institution. Authorisation was obtained from the school directors, the students’ guardians, and the students themselves assented for participation in the research. The assessments were divided into three sessions, held at the school in a suitable location, and were carried out individually or in groups, following the application guidelines of each test. At the conclusion of the analysis, individual data and the overall research results were shared with the respective guardians of the students. The application followed a fixed order, first session: WASI or Raven’s Coloured Progressive Matrices (according to the student’s age at the time of the test), FVF, Hayling Test, Digit Tasks, and Reading (from TDE-II); second session: individual (1st to 3rd grades) and collective (from 4th grade) application of the Writing Subtest (TDE-II); third session: individual (1st to 3rd grades) and collective (from 4th grade) application of the Arithmetic Subtest (TDE-II).

### 2.4. Data Analysis Procedures

Descriptive statistics (frequency, percentage, mean, and standard deviation) were used to analyse sociodemographic data and characterise the participants. Additionally, mean and standard deviation were employed to describe data across age groups or school grades in the pre-pandemic period (based on instrument norms) and the post-pandemic period (based on data collected in this study), considering each task and dependent variable separately [36].

To examine potential differences between the sample in this study and the normative samples established in the pre-pandemic period, as described in the test manuals, one-sample *t*-tests were conducted [36]. Given the small sample size for each school grade and age, comparative analyses also included confidence intervals for the differences, estimated using the bootstrapping resampling procedure (1000 resamplings, BcA—bias-corrected and accelerated, with a 95% confidence interval). The BcA bootstrapping method was used to mitigate issues related to the lack of normal data distribution due to the small sample size, improve the accuracy of confidence intervals, and correct biases and asymmetries in the distribution of estimates [37].

Furthermore, effect size was calculated using Cohen’s d [38] to quantify the magnitude of the differences, applying the following criteria: *d* > 0.80 = large effect; *d* = 0.50 = medium effect; *d* = 0.20 = small effect. A significance level of *p* < 0.05 was considered statistically significant. All analyses were conducted using IBM SPSS Statistics software (version 27.0).

## 3. Results

Table 2, Table 3, Table 4, Table 5, Table 6, Table 7, Table 8 and Table 9 present the participants’ performance in terms of descriptive statistics (mean and standard deviation), as well as the normative data, and the *t*-test results. The executive function measures are presented by age, while the academic performance measures are presented by school year because the respective test manuals grouped the normative sample data this way.

Regarding working memory performance comparisons, assessed using the Digit Span Test (see Table 2), significant differences were observed at all ages except for participants aged 8 and 9 years. The confidence interval in the bootstrap analysis supported these findings. Notably, students aged 6 and 7 years performed significantly better than the normative samples of the instrument. The effect size in these comparisons ranged from large to medium, respectively. In contrast, students aged 10 to 14 years performed significantly worse than the pre-pandemic norms, with effect sizes ranging from small to medium.

Regarding verbal fluency, when comparing the current mean with the normative mean of the Verbal Fluency Test (Table 3), statistically significant differences were observed in the results of participants aged 9 to 14 years. These differences were corroborated by the confidence interval in the bootstrap analysis, with the current sample showing poorer performance compared to pre-pandemic norms across all ages. In all those cases, the effect sizes ranged from medium to large, with the most pronounced differences observed in students aged 11 to 14 years. It is important to note that, for verbal fluency measures, the norms for 12-year-olds were used to compare participants aged 13 and 14 due to the absence of norms for this age group. Even when compared to younger age norms, the present sample of 13- and 14-year-olds performed worse than the normative mean.

In terms of errors on the Hayling Test (see Table 4), which measures cognitive flexibility and inhibitory control, statistically significant differences were observed between participants aged 7, 9, 10, 11, 12, 13, and 14, with the present study sample showing lower performance (i.e., higher number of errors) compared to pre-pandemic normative means. The effect size also ranged from medium to large, indicating the relevance of the differences. In the group of 6-year-old participants, although the *p*-value (*p* = 0.056) did not reach the conventional statistical significance threshold (*p* < 0.05); the bootstrap analysis, combined with a large effect size, suggests potential practical significance. As mentioned previously, this result indicates that the small sample size (n = 8) may have compromised the statistical power of the analysis.

Regarding the comparisons involving the analysis of reaction time from the Hayling B Test, which also provides measures of cognitive flexibility and inhibitory control, the results revealed significant differences in the participants aged 10 to 12 years (Table 5). In these groups, the participants presented faster performance compared to the normative means. These results were corroborated by the confidence intervals obtained in the bootstrap analyses. In these comparisons, the effect sizes ranged from medium to large. For the 6-year-old group, although the *p* value (*p* = 0.171) did not reach the conventional level of statistical significance (*p* < 0.05), the analysis of the bootstrap confidence interval and the average effect size indicate the presence of a relevant difference. This discrepancy may reflect a Type II error, possibly due to the small sample size (n = 8), which compromises the statistical power of the analysis [39].

Writing ability was assessed by the Writing Subtest of the TDE-II. As shown in Table 6, students from the fifth to eighth grades showed statistically significant differences between the means, with weaker post-pandemic performance. These results were corroborated by the confidence intervals of the bootstrap analysis. The effect size ranged from medium to large in these comparisons.

For the word reading score, assessed using the TDE-II Reading Subtest, students from the fifth to eighth grades performed significantly lower than expected when compared to pre-pandemic norms, which was corroborated by the bootstrap analysis confidence interval and the observed mean effect sizes (Table 7). In the fourth grade, although the *p*-value (*p* = 0.058) did not reach the conventional level of statistical significance, the bootstrap analysis, combined with a robust effect size, indicates a potential practical significance. This finding suggests that the sample size limitation may have influenced the results of the t-test analysis and, therefore, suggests that the bootstrapping method was necessary to confirm the results. Regarding the execution time in this reading subtest (Table 8), only the second grade performed faster compared to the normative mean, a result that was corroborated by the bootstrap analysis. The effect size of this comparison was large.

Regarding arithmetic, a skill assessed by the Arithmetic Subtest of the TDE-II, eighth and ninth grade students performed worse than the pre-pandemic normative averages, a result corroborated by the confidence interval obtained in the bootstrap analysis, with large effect sizes for both groups (Table 9). In the group of fourth grade students, although the *p*-value (*p* = 0.066) did not reach the conventional threshold of statistical significance (*p* < 0.05), both the bootstrap confidence interval and the effect size suggest the presence of practical relevance, with lower performance in the current sample. This finding indicates that the reduced sample size may have compromised the statistical power, making it difficult to detect significant differences even in the face of a trend of lower performance than the standardised data. Likewise, the groups of first, second, and fifth grade students presented non-significant *p*-values; however, subsequent analyses—through bootstrap confidence intervals and effect sizes (d ranging from 0.410 to 0.484)—suggest that the sample limitation may have masked the practical relevance of the results [38], which indicate superior performances in the current sample compared to pre-pandemic norms.

Table 10 presents a comparative summary of the significant results obtained in this study when comparing the post-pandemic data with pre-pandemic normative data. Executive function measures are presented by age, while academic performance measures are presented by school year, since the respective test manuals grouped the normative data of the sample in this way. It should be noted that for score measures, the higher the value, the better the performance (top of the table). Conversely, for error and time measures, the higher the value, the worse the performance (bottom of the table).

## 4. Discussion

The present study compared child cognition (short-term verbal memory, working memory, verbal fluency, cognitive flexibility, inhibitory control, processing speed) and cognitive achievement (reading, writing, and arithmetic) performance after the COVID-19 pandemic with the norms established for these variables before, by age and school grade. Specifically, our preliminary results showed EF and academic losses across the first to ninth school years in Brazil, a population underrepresented in educational neuroscience literature [4].

Overall, we have found that in the majority of the tests, children showed lower scores after the pandemic than before, especially between 10 and 13 years (i.e., between the fifth and eighth grades). No difference was observed at age 8 (i.e., third grade). This general result is in line with previous studies showing that the suspension of in-person education impacted negatively on child cognitive development and academic achievement [1,2,3,4,5,12,40] and also with studies conducted in Brazil [14,17]. Our data do not let us to explain the reasons for the findings, but it might be worth mentioning that limitations such as the lack of computers or internet access as well as the difficulty understanding school assignments jointly with the problems to find someone to help with schoolwork were reported in Brazil as potential explanations for the decrease in achievement [15,16]. However, the findings showing a higher number of differences in the oldest children and the higher grades contrast with studies conducted in the country. For example, Alves and collaborators [17] analysed children across the second to fifth grades of elementary school and found an impact of class suspension on reading fluency only in the second grade children, but they analysed a sample coming from private high socioeconomic status schools. Instead, the schools in our study were from middle-to-low socioeconomic status contexts. It has been proposed that older students, in the final years of elementary school or in high school, seem to be more familiar with technology and, therefore, more autonomous, taking most advantage from virtual classes [18,19,20]. However, if socioeconomic conditions impede access to computers or to the internet, technology familiarity would not have any effect. Future studies should analyse how the family socioeconomic level can interact with the student’s age to produce different impacts on reading.

One strength of our study is the number of tests implemented, and it is also an example of how educational neuroscience has contributed to a very detailed evaluation of the impact of the pandemic. In this preliminary analysis of the findings for each test, our results revealed a significant decline in the average verbal fluency performance, especially among participants aged 9 to 14 years, and remarkably, when compared to younger age norms. This result aligns with investigations conducted in Brazil, which reported an increase in complaints of difficulties related to EF among students in the early years of elementary school [25]. Furthermore, Ribeiro, Celeste, and Reis [26] found that 48.72% of children enrolled in the first and second years of elementary school, after the pandemic period, presented scores indicating warnings or deficits in verbal fluency. In sum, our results suggest that class suspension is associated with lower vocabulary (number of words acquired by individuals).

Regarding the scores obtained in working memory, we found a similar trend: a significant decline was observed when comparing the current average with the normative average, particularly among older students. The decrease in working memory associated with the pandemic has been observed in previous studies [26,41], and it has been suggested that it might be linked with the anxiety produced by the lockdown [41]. Conversely, the fact that students aged 6 and 7 achieved scores higher than the pre-pandemic norm contrasted with the findings of the study conducted in Brazil by Ribeiro, Celeste, and Reis [26], whose results indicated that 62.4% of the children in the first and second years of elementary school showed scores indicative of deficit in working memory. Remarkably, a study conducted in Ethiopia compared test results applied to groups before and after school closures, demonstrating higher scores in working memory and non-verbal intelligence in the group assessed after the return to in-person school activities [42]. Future studies should clarify the reason for these differences.

In relation to inhibitory control, with the exception of 8-year-old students, all other participants had a higher number of errors than the expected normative average. Conversely, they showed faster performance than expected according to the guidelines established before the pandemic, but this is not interpreted as an improvement given that the higher speed is associated with a worse achievement in the task. The high number of errors is in line with previous evidence showing similar results [25]. Daily screen use has been suggested as one of the factors decreasing inhibition in American school-age children [42], but in Brazil, only 31% of public elementary school students had a computer/tablet and broadband at home [43], and therefore more evidence is needed to explain the relationship between these factors in Global South countries.

Regarding educational outcomes, our results generally replicate previous findings [2]: the pandemic negatively affected academic achievement across various educational areas, mainly in the fifth and eighth grades. Studies [3,4] indicate that prolonged educational disruption has resulted in attenuated learning gains. Further, if that impact was significant in countries from the Global North [2], and the most remarkable effects were for those already at risk for educational disparities [6,44], we might expect a bigger effect in countries from the Global South such as Brazil, which is actually what we found, and what is in line with recent meta-analysis results [4]. Among the factors explaining those effects, the direct lack of educational opportunities, as well as the stressors produced by the lockdown, were suggested as important variables [12].

Specifically, we found that in reading and writing tests, children had lower scores after the pandemic, between the fifth and eighth grades. It is important to emphasise that these students, during the pandemic, were in the process of literacy development and/or in the early years of elementary education and that a recent meta-analysis [3] has suggested that younger students were more affected by school closure. Our results, although preliminary, corroborate the ones obtained by Santos [21], which revealed significantly lower performance compared to pre-pandemic standards among second, fourth, and fifth grade students from high socioeconomic status schools, showing that in this area the pandemic impacted across all economic levels. We have also observed that second grade students were faster post-pandemic, although this result might have been due to particularities of the evaluated sample such as the homogeneity (the standard deviation is small in comparison with standard deviation of first year students as well as with the norm). However, the general trend indicated that the pandemic affected negatively reading and writing achievements. Brazilian studies conducted with children in the early years of elementary school also highlighted learning gaps related to reading comprehension and fluency [17]. Regarding writing, it has been suggested that the use of digital platforms did not allow for the stimulation of students’ motor skills [45]. This situation had already been anticipated by authors who discussed the potential risks associated with low-quality education resulting from remote learning [12] and by studies showing similar results with big sample sizes [2,42].

Regarding arithmetic skills, the results are the opposite: at some grades, they improved after the pandemic, but in other ones, they decreased. Specifically, at the first, second, and fifth grades, children improved after the pandemic. Instead, the fourth, eighth, and ninth grade students showed a significant decline in performance when compared to pre-pandemic normative averages. This last result agrees with the one obtained by Santos [21], which identified a decline in the academic performance of students in the third, fourth, and fifth grades, when comparing their results to the instrument’s normative standards. However, the result of the improvement for the first years is unexpected, and also contradictory with studies involving big samples [2,46]. Future studies should confirm our results.

In summary, the results obtained in the assessment of academic skills are generally consistent with data from national [15] and international [3,4,16] educational evaluations, which indicated a decline in student performance. This phenomenon is closely associated with the impact of the COVID-19 pandemic, particularly in countries such as Brazil which kept educational institutions closed for extended periods and faced significant challenges in implementing remote learning [14]. In the face of humanitarian crises and natural disasters, the disruption of access to the educational system can lead to long-term consequences, even with the implementation of compensatory measures [44].

In general, it was observed that students who were in the early years of primary education during the pandemic—especially those in the second to fifth grades (currently in the fifth to eighth grades)—showed the most compromised performance in language skills (reading and writing) compared to pre-pandemic norms, a finding consistent with previous studies [3]. They also tended to perform worse in specific executive function skills, such as working memory and verbal fluency. The contribution of this work was to add data on the effects of the pandemic on children from the Global South, having been pointed out as a need in the field [4]. The strength is that the administration of various tests led us to explain in high detail the impact, according to age or grade, as well as to the cognitive or educational process.

However, this study also has limitations. One limitation is the small sample size. Although statistical procedures (such as bootstrapping) were applied to mitigate issues related to the limited sample, the results should be taken as preliminary. Also, it is important to emphasise that the results are not generalisable, due to the use of a convenience sample. Another limitation concerns the type of design employed. When comparing the sample results to pre-pandemic normative data, the interpretation of findings must be approached with caution, as there may be variations in participants’ characteristics. Although pre-post longitudinal designs with larger samples would have been more appropriate, they were not possible in this case, as there were no data taken when the school closure started. Therefore, we chose a design previously used [21] because it was the better option with the available information. It is equally relevant to consider the potential diversity of the schools participating in the study, in terms of different approaches to the reception and management of the learning process during the closure of institutions and the adoption of hybrid teaching, including the availability of internet and electronic devices. Nonetheless, given the unavailability of longitudinal data with a large number of participants assessed prior to the pandemic, the current design still provides valuable insights into potential areas of performance decline.

## 5. Conclusions

Despite the limitations, the present study makes an important contribution by providing preliminary information on executive functioning and the academic skills of Brazilian students in the post-COVID-19 pandemic period. Considering the timing of data collection, after two years of the return to in-person classes, a significant gap was identified in the evaluated skills. Such a finding reinforces the need to develop strategies and educational measures aimed at reducing the emerging learning gaps due to the pandemic. Although our results are not generalisable, the identification of performance declines remains critical for guiding educational policies. In fact, according to UNICEF [1], the assessment of learning is essential for guiding effective recovery strategies, ensuring resources are allocated where they are most needed, and helping teachers adapt instruction to address students’ specific gaps. Then, by identifying specific cognitive areas and school years with poorer performance, targeted intervention can be developed for these students. This is crucial to ensure that potential deficits resulting from remote learning are addressed and solved, rather than becoming long-term obstacles in students’ academic trajectories.

## Figures and Tables

**Table 1:** Participants’ school year at the time of data collection and during the pandemic period.

School Year at the Time of Data Collection	Data Collection Conducted in 2023	Data Collection Conducted in 2024	School Year in 2020 (1st Year of the Pandemic)	School Year in 2021 (2nd Year of the Pandemic)	School Year Overall During the Pandemic
1st grade	X		Preschool	Pre-kindergarten	Preschool–Pre-kindergarten
2nd grade	X		Pre-kindergarten	Kindergarten	Pre-kindergarten–Kindergarten
3rd grade	X		Kindergarten	1st grade	Kindergarten–1st grade
4th grade	X		1st grade	2nd grade	1st–2nd grade
5th grade	X		2nd grade	3rd grade	2nd–3rd grade
6th grade		X	2nd grade	3rd grade	2nd–3rd grade
7th grade		X	3rd grade	4th grade	3rd–4th grade
8th grade		X	4th grade	5th grade	4th–5th grade
9th grade	X		6th grade	7th grade	6th–7th grade
9th grade		X	5th grade	6th grade	5th–6th grade

**Table 2:** Student’s *t*-test of the effect of group (sample from this study compared to the normative sample) on scores in a measure of working memory (DST and Digit Span Subtest from WISC-IV).

Age (n)	Pre-Pandemic Standard ^a^	Post-Pandemic Performance	t	df	Sig ^c^	Mean Difference	BCa 95% Confidence Interval of the Difference ^d^		d
	Mean (SD)	Mean (SD)					Lower	Higher	
6 (n = 8)	6.82 (------) ^b^	9.00 (2.45)	2.517	7	0.040	2.180	0.305 ^e^	3.680 ^e^	0.890
7 (n = 18)	8.22 (------) ^b^	9.28 (1.99)	2.250	17	0.038	1.058	0.113	1.947	0.530
8 (n = 12)	9.76 (------) ^b^	9.58 (1.73)	−0.354	11	0.730	−0.177	−0.927	0.657	−0.102
9 (n = 26)	10.94 (------) ^b^	11.19 (2.62)	0.490	25	0.628	0.252	−0.517	1.022	0.096
10 (n = 27)	11.62 (------) ^b^	10.22 (2.04)	−3.553	26	<0.001	−1.398	−2.139	−0.546	−0.684
11 to 13 ^f^ (n = 69)	14.67 (------) ^b^	12.71 (2.22)	−7.343	68	<0.001	−1.960	−2.554	−1.308	−0.884
14 (n = 18)	16.19 (------) ^b^	14.71 (3.47)	−2.475	17	0.024	−2.023	−3.134	−0.801	−0.583

Note: ^a^ Reference of the origin of the standard [28,29]; ^b^ the standard deviation was not reported by the authors; ^c^ significance of the *t*-test for one sample (2 tails); ^d^ confidence interval of the difference for bootstrap comparison based on 1000 bootstrap samples; d = Cohen’s d (effect size); ^e^ Based on 999 samples. ^f^ The ages of 11 to 13 are grouped because the instrument’s manual presents the averages this way.

**Table 3:** Student’s *t*-test of the effect of group (sample from this study compared to the normative sample) on the score in the Free Fluency Verbal measure (FVL).

Age (n)	Pre-Pandemic Standard ^a^	Post-Pandemic Performance	t	df	Sig ^b^	Mean Difference	BCa 95% Confidence Interval of the Difference ^c^		d
	Mean (SD)	Mean (SD)					Lower	Higher	
6 (n = 8)	23.68 (11.39)	24.00 (3.93)	0.230	7	0.824	0.320	−2.180	2.445	0.081
7 (n = 18)	28.10 (13.12)	33.22 (12.86)	1.690	17	0.109	5.122	−1.155	11.844	0.398
8 (n = 12)	31.18 (13.52)	27.75 (9.35)	−1.270	11	0.230	−3.430	−7.597	1.320	−0.367
9 (n = 26)	40.28 (14.25)	32.81 (13.90)	−2.742	25	0.011	−7.472	−11.742	−2.731	−0.538
10 (n = 27)	46.00 (12.64)	36.74 (13.63)	−3.530	26	0.002	−9.259	−13.556	−4.520	−0.679
11 (n = 28)	50.47 (20.11)	33.03 (13.16)	−7.008	27	<0.001	−17.434	−22.357	−11.937	−1.324
12 (n = 23)	52.36 (16.35)	32.74 (13.41)	−7.013	22	<0.001	−19.621	−25.113	−14.268	−1.462
13 to 14 ^e^ (n = 36)	52.36 (16.35)	32.83 (14.42)	−8.124	35	<0.001	−19.527	−24.462	−13.356	−1.354

Note: ^a^ Reference of the source of the standard [30]; ^b^ significance of the one-sample *t*-test (2 tails); ^c^ confidence interval of the difference for bootstrap comparison based on 1000 bootstrap samples; d = Cohen’s d (effect size).^e^ the ages of 13 and 14 are grouped because the instrument’s manual presents the averages this way.

**Table 4:** Student’s *t*-test of the effect of group (sample of this study compared to the normative sample) on error score in measure of inhibitory control/cognitive flexibility (Hayling B).

Age (n)	Pre-Pandemic Standard ^a^	Post-Pandemic Performance	t	df	Sig ^b^	Mean Difference	BCa 95% Confidence Interval of the Difference ^c^		d
	Mean (SD)	Mean (SD)					Lower	Higher	
6 (n = 8)	7.25 (1.39)	8.00 (0.93)	2.291	7	0.056	0.750	0.250 ^d^	1.250 ^d^	0.810
7 (n = 18)	5.95 (1.70)	7.27 (1.45)	3.892	17	<0.001	1.328	0.661	2.050	0.917
8 (n = 12)	5.58 (1.55)	6.25 (2.18)	1.065	11	0.310	0.670	−0.413	1.670	0.307
9 (n = 26)	4.65 (2.01)	6.50 (1.39)	6.773	25	<0.001	1.850	1.388	2.275	1.328
10 (n = 27)	4.60 (2.14)	6.33 (1.92)	4.687	26	<0.001	1.733	1.184	2.326	0.902
11 (n = 28)	4.57 (1.79)	5.71 (2.27)	2.662	27	0.013	1.144	0.359	1.859	0.503
12 (n = 23)	3.78 (1.59)	4.65 (1.37)	3.056	22	0.006	0.872	0.437	1.307	0.637
13 to 14 (n = 36)	4.01 (2.28)	5.86 (3.06)	3.626	35	<0.001	1.851	0.879	2.796	0.604

Note: ^a^ Reference of the source of the standard [31,32]; ^b^ significance of the one-sample *t*-test (2 tails); ^c^ confidence interval of the difference for bootstrap comparison based on 1000 bootstrap samples; ^d^ Based on 999 samples.; d = Cohen’s d (effect size).

**Table 5:** Student’s *t*-test of the effect of group (sample of this study compared to the normative sample) on time score (seconds) in a measure of inhibitory control/cognitive flexibility (Hayling B).

Age (n)	Pre-Pandemic Standard ^a^	Post-Pandemic Performance	t	df	Sig ^b^	Mean Difference	BCa 95% Confidence interval of the difference ^c^		d
	Mean (SD)	Mean (SD)					Lower	Higher	
6 (n = 8)	57.39 (17.76)	81.00 (43.77)	1.526	7	0.171	23.610	1.543	52.052	0.539
7 (n = 18)	42.35 (18.58)	54.55 (33.57)	1.542	17	0.141	12.206	−0.701	26.788	0.364
8 (n = 12)	54.16 (16.37)	48.25 (20.94)	−0.977	11	0.349	−5.910	−18.430	7.007	−0.282
9 (n = 26)	41.18 (17.35)	39.69 (21.20)	−0.358	25	0.724	−1.488	−8.861	7.578	−0.070
10 (n = 27)	43.79 (17.82)	30.24 (13.32)	−5.283	26	<0.001	−13.549	−17.805	−8.935	−1.017
11 (n = 28)	41.08 (10.49)	28.98 (18.10)	−3.535	27	0.001	−12.092	−17.828	−4.595	−0.668
12 (n = 23)	36,23 (9.53)	26.96 (8.96)	−4.962	22	<0.001	−9.270	−12.208	−6.078	−1.035
13 to 14 (n = 36)	52.95 (19.21)	41.04 (24.68)	1.170	35	0.250	4.813	−3.710	14.268	0.195

Note: ^a^ Reference of the source of the standard [31,32]; ^b^ significance of the one-sample *t*-test (2 tails); ^c^ confidence interval of the difference for bootstrap comparison based on 1000 bootstrap samples; d = Cohen’s d (effect size).

**Table 6:** Student’s *t*-test of the effect of group (sample of this study compared to the normative sample) on writing measure (Writing Subtest TDE-II).

School Year (n)	Pre-Pandemic Standard ^a^	Post-Pandemic Performance	t	df	Sig ^b^	Mean Difference	BCa 95% Confidence Interval of the Difference ^c^		d
	Mean (SD)	Mean (SD)					Lower	Higher	
1 (n = 15)	5.18 (8.49)	5.53 (5.48)	0.249	14	0.807	0.353	−1.647	2.820	0.064
2 (n = 18)	14.91 (10.67)	15.55 (9.53)	0.287	17	0.774	0.646	−3.577	4.979	0.068
3 (n = 18)	25.70 (7.31)	22.88 (9.85)	−1.210	17	0.243	−2.811	−8.422	2.075	−0.285
4 (n = 23)	27.76 (7.44)	28.00 (7.77)	0.148	22	0.884	0.240	−3.462	3.558	0.031
5 (n = 25)	10.62 (6.88)	5.84 (5.30)	−4.505	24	<0.001	−4.780	−6.474	−2.846	−0.901
6 (n = 22)	14.41 (7.38)	10.45 (7.86)	−2.360	21	0.028	−3.955	−7.223	−0.365	−0.503
7 (n = 21)	19.18 (7.86)	11.47 (8.15)	−4.327	20	<0.001	−7.704	−11.228	−3.703	−0.944
8 (n = 19)	20.81 (7.41)	11.31 (6.51)	−6.352	18	<0.001	−9.494	−11.932	−7.073	−1.457
9 (n = 17)	21.52 (8.67)	17.41 (10.10)	−1.677	16	0.113	−4.108	−8.814	0.951	−0.407

Note: ^a^ Reference of the source of the standard [33]; ^b^ significance of the one-sample *t*-test (2 tails); ^c^ confidence interval of the difference for bootstrap comparison based on 1000 bootstrap samples; d = Cohen’s d (effect size).

**Table 7:** Student’s *t*-test of the effect of group (sample of this study compared to the normative sample) on reading measure, raw score (TDE reading).

School Year (n)	Pre-Pandemic Standard ^a^	Post-Pandemic Performance	t	df	Sig ^b^	Mean Difference	BCa 95% Confidence Interval of the Difference ^c^		d
	Mean (SD)	Mean (SD)					Lower	Higher	
1 (n = 15)	8.55 (13.38)	12.00 (10.95)	1.220	14	0.243	3.450	−1.408	8.441	0.315
2 (n = 18)	23.62 (13.96)	26.77 (11.01)	1.216	17	0.241	3.158	−2.251	7.658	0.287
3 (n = 18)	32.84 (4.81)	31.00 (8.91)	−0.876	17	0.393	−1.840	−7.206	1.581	−0.206
4 (n = 23)	34.29 (4.75)	33.17 (2.67)	−2.002	22	0.058	−1.116	−2.290	−0.203	−0.417
5 (n = 25)	28.06 (4.29)	25.76 (4.17)	−2.754	24	0.011	−2.300	−3.740	−0.740	−0.551
6 (n = 22)	28.78 (4.07)	24.72 (5.86)	−3.241	21	0.004	−4.053	−6.529	−1.871	−0.691
7 (n = 21)	29.69 (3.08)	27.23 (3.97)	−2.828	20	0.010	−2.452	−4.261	−0.909	−0.617
8 (n = 19)	30.50 (2.42)	27.26 (5.39)	−2.616	18	0.018	−3.237	−5.564	−1.278	−0.600
9 (n = 17)	30.52 (2.01)	29.11 (3.82)	−1.513	16	0.150	−1.402	−3.226	0.294	−0.367

Note: ^a^ Reference of the source of the standard [33]; ^b^ significance of the one-sample *t*-test (2 tails); ^c^ confidence interval of the difference for bootstrap comparison based on 1000 bootstrap samples; d = Cohen’s d (effect size).

**Table 8:** Student’s *t*-test of the effect of group (sample from this study compared to the normative sample) on reading measure, time (TDE reading).

School Year (n)	Pre-Pandemic Standard ^a^	Post-Pandemic Performance	t	df	Sig ^b^	Mean Difference	BCa 95% Confidence Interval of the Difference ^c^		d
	Mean (SD)	Mean (SD)					Lower	Higher	
1 (n = 15)	189.33 (139.39)	198.40 (124.89)	0.281	14	0.783	9.070	−63.967	97.226	0.073
2 (n = 18)	155.44 (152.83)	103.94 (50.49)	−4.327	17	<0.001	−51.495	−70.753	−30.051	−1.020
3 (n = 18)	71.68 (43.35)	90.44 (71.99)	1.106	17	0.284	18.764	−9.349	57.208	0.261
4 (n = 23)	49.38 (19.01)	54.43 (17.79)	1.362	22	0.187	5.054	−1.946	13.318	0.284
5 (n = 25)	97.48 (27.37)	108.76 (55.02)	1.025	24	0.315	11.285	−4.975	30.985	0.205
6 (n = 22)	89.21 (29.80)	96.13 (55.26)	0.587	21	0.563	6.920	−11.289	28.044	0.125
7 (n = 21)	77.13 (20.70)	81.83 (35.04)	0.615	20	0.546	4.701	−11.251	20.655	0.134
8 (n = 19)	70.88 (18.51)	75.64 (25.45)	0.816	18	0.425	4.765	−5.734	15.144	0.187
9 (n = 17)	60.11 (15.27)	75.70 (44.32)	1.451	16	0.166	15.594	−3.120	38.858	0.352

Note: ^a^ Reference of the source of the standard [33]; ^b^ significance of the one-sample *t*-test (2 tails); ^c^ confidence interval of the difference for bootstrap comparison based on 1000 bootstrap samples; d = Cohen’s d (effect size).

**Table 9:** Student’s *t*-test of the effect of group (sample of this study compared to the normative sample) on arithmetic measure, raw score (arithmetic TDE).

School Year (n)	Pre-Pandemic Standard ^a^	Post-Pandemic Performance	t	df	Sig ^b^	Mean Difference	BCa 95% Confidence Interval of the Difference ^c^		d
	Mean (SD)	Mean (SD)					Lower	Higher	
1 (n = 15)	8.07 (3.81)	9.40 (2.74)	1.876	14	0.082	1.330	0.063	2.530	0.484
2 (n = 18)	12.91 (4.27)	14.55 (3.71)	1.880	17	0.077	1.646	0.034	3.090	0.443
3 (n = 18)	19.67 (4.10)	18.88 (3.77)	−0.879	17	0.392	−0.781	−2.614	0.997	−0.207
4 (n = 23)	23.73 (3.35)	22.08 (4.06)	−1.938	22	0.066	−1.643	−3.121	−0.252	−0.404
5 (n = 25)	25.00 (5.18)	26.48 (3.60)	2.052	24	0.051	1.480	0.040	2.800	0.410
6 (n = 22)	8.37 (6.03)	7.95 (5.13)	−0.380	21	0.708	−0.415	−2.188	1.572	−0.081
7 (n = 21)	12.75 (7.01)	10.09 (6.01)	−2.022	20	0.057	−2.655	−5.309	0.332	−0.441
8 (n = 19)	16.26 (9.48)	7.84 (5.03)	−7.286	18	<0.001	−8.418	−10.049	−6.418	−1.672
9 (n = 17)	16.00 (8.18)	8.70 (6.66)	−4.510	16	<0.001	−7.294	−10.052	−4.059	−1.094

Note: ^a^ Reference of the source of the standard [33]; ^b^ significance of the one-sample *t*-test (2 tails); ^c^ confidence interval of the difference for bootstrap comparison based on 1000 bootstrap samples; d = Cohen’s d (effect size).

**Table 10:** Comparative summary of the significant results comparing post-pandemic data with pre-pandemic normative data for different ages and school years across various indices of the instruments that assess executive function skills and academic performance.

	**AGE**								
**RAW SCORE**	6	7	8	9	10	11	12	13	14
Working Memory—(Digit)	> *	> *			<	<	<	<	<
FVerbal Fluency (FVL)				<	<	<	<	<	<
	**SCHOOL YEAR**								
**RAW SCORE**	1	2	3	4	5	6	7	8	9
Writing					<	<	<	<	
Reading				< *	<	<	<	<	
Arithmetic	> *	> *		< *	> *			<	<
	**AGE**								
**TIME** **∕** **ERROR**	6	7	8	9	10	11	12	13	14
Hayling B—time	< *				>	>	>		
Hayling B—error	< *	<		<	<	<	<	<	<
	**SCHOOL YEAR**								
**TIME** **∕** **ERROR**	1	2	3	4	5	6	7	8	9
Reading-time		>							

< indicates that the current sample performed statistically worse than the normative sample (*p* < 0.05). > indicates that the current sample performed statistically better than the normative sample (*p* < 0.05). * Score whose bootstrap analysis suggested potential statistical significance.

## Data Availability

The raw data supporting the conclusions of this article will be made available by the authors on request.

## Abbreviations

The following abbreviations are used in this manuscript:

EF	Executive functions
INEP	Instituto Nacional de Estudos e Pesquisa Educacionais Anísio Teixeira
PISA	Programme for International Student Assessment
SaeB	Basic Education Assessment System
IN	Inhibition
WM	Working memory
CF	Cognitive flexibility
IDEB	Basic Education Development Index
DST	Digit Span Task
WISC IV	Wechsler Intelligence Scale for Children
FVL	Free Verbal Fluency
HT	Hayling Test
CHT	Children’s Hayling Test
AHT	Adult’s Hayling Test
TDE II	School Performance
CPM	Raven’s Coloured Progressive Matrices
WASI	Wechsler Abbreviated Scale of Intelligence
